# Mapping soil suitability using phenological information derived from MODIS time series data in a semi-arid region: A case study of Khouribga, Morocco

**DOI:** 10.1016/j.heliyon.2024.e24101

**Published:** 2024-01-08

**Authors:** Maryem Ismaili, Samira Krimissa, Mustapha Namous, Kamal Abdelrahman, Abdelghani Boudhar, Mohamed Edahbi, Youssef Lebrini, Abdelaziz Htitiou, Soufiane Maimouni, Tarik Benabdelouhab

**Affiliations:** a Data4Earth Laboratory, Faculty of Sciences and Technology, Sultan Moulay Slimane University, Beni Mellal, Morocco; b Department of Geology & Geophysics, College of Science, King Saud University, Riyadh, 11451, Saudi Arabia; c Superior School of Technology Fkih Ben Salah, Sultan Moulay Slimane University, Morocco; d University of Quebec in Abitibi-Témiscamingue (UQAT), Rouyn-Noranda, J9X 5E4, QC, Canada; e Laboratory of Applied Geology, Geoinformatic and Environment, Department of Geology, Faculty of Sciences Ben M’sik, Hassan II University of Casablanca, B.P. 7955, Sidi Othmane, Casablanca, Morocco; f National Agronomic Research Institute, Rabat, Morocco

**Keywords:** Precision agriculture, Soil mapping, Multi-criteria analysis (MCAD), Large integral (LINTG), NDVI

## Abstract

To address the increasing global demand for food, it is crucial to implement sustainable agricultural practices, which include effective soil management techniques for enhancing productivity and environmental conditions. In this regard, a study was conducted to assess the efficacy of utilizing phenological metrics derived from satellite data in order to map and identify suitable agricultural soil within a semi-arid region. Two distinct methodologies were compared: one based on physicochemical soil parameters and the other utilizing the phenological response of vegetation through the application of the Normalized Difference Vegetation Index (NDVI) Modis-time series. The study findings indicated that the NDVI-based approach successfully identified a specific class of soil suitability for agriculture (referred to as S1) that could not be effectively mapped using the multi-criteria analysis (MCAD) method relying on soil physicochemical parameters. This S1 class of soil suitability accounted for approximately 5 % of the total study area. These outcomes suggest that phenological-based approaches offer greater potential for spatio-temporal monitoring of soil suitability status compared to MCAD, which heavily relies on discrete observations and necessitates frequent updates of soil parameters. The approach developed to map the soil-suitability is a valuable tool for sustainable agricultural development, and it can play an effective role in ensuring food security and conducting a land agriculture assessment.

## 1 Introduction

Sustainable agricultural development is a crucial objective in all regions, regardless of their level of development. The primary objective of sustainable agriculture is to maintain the balance between inherent soil resources and crop requirements while optimizing resources to achieve long-term productivity [1]. Soil studies have gained significant attention in recent decades due to the severe consequences of soil degradation. Proper exploitation of soils as a valuable natural resource is necessary to meet the food requirements of a growing population. It is, therefore, essential to manage these resources sustainably, taking into account climate change, on-ground regional characteristic situations. The degradation of fertile land in arid and semi-arid regions is attributed to a combination of climate change and human activities. These factors have led to partial or complete deterioration of the land, resulting in the loss of the least productive and poorest quality soil. This degradation negatively impacts the agricultural potential and productivity of the affected areas, exacerbating the challenges faced by communities in these regions. Furthermore, having a comprehensive understanding of the current state of soil degradation is essential for developing policies that focus on environmental preservation, restoration, and sustainable management. Additionally, it is important to recognize that soil erosion is a natural process influenced by various factors, including rainfall, soil properties, topographic features, and hydrogeological systems. However, human activities have significantly escalated erosion rates globally, surpassing natural levels by a magnitude of 10–40 [[2], [3], [4]]. Assessing soil suitability can aid in optimizing the monitoring of agricultural areas and evaluating the land's agronomic potential [5,6]. Overuse and degradation are among the most critical factors that contribute to soil weakness [6]. Monitoring soil quality is a crucial process for predicting the suitability of land use types (LUTs) in a given area and provides a sound basis for land use planning, especially in developing countries (Herzberg et al., 2019). Soil suitability maps aim to offer practical solutions regarding the selection of land use by providing information on their agronomic potential [7,8]. Various methods and models have been used for soil suitability mapping, such as Linear Combination [9], Simple Limitation [10], fuzzy-logic modeling [11], the use of Artificial Neural Networks [12], Remote sensing [[13], [14], [15]], Machine learning [16], Multi-Criteria Decision Analysis (MCDA), Multi-Criteria Evaluation (MCE) [17,18], and the Analytical Hierarchy Process (AHP) [19,20]. Although AHP has some limitations, MCE and MCDA are still the most commonly used methods for soil evaluation, particularly on a small scale [21]. However, these methods are time-consuming, involve expensive procedures in sampling and ground surveying of data, and fail to detect the spatiotemporal variability in soil properties that result mainly from anthropogenic activities such as mining and quarrying, soil salinization, and erosion processes.

Remote sensing tools can overcome the limitations of classical soil suitability mapping by providing an alternative method across various spatio-temporal scales. This can be achieved by using satellite-derived vegetation response to soil, climate, and agricultural techniques' conditions, which offers a potential alternative proxy of soil suitability [[22], [23], [24], [25]]. Although vegetation can often obstruct soil visibility for much of the year, multiple measurements of vegetation development can be used to indirectly assess soil potential. Reflectance data from optical satellites can be used to generate a set of vegetation indices and derived phenological parameters, which are widely used in monitoring the earth's surface [4,15,22,[26], [27], [28]]. Phenological parameters can discriminate the impact of soil suitability on vegetation development from other confounding factors. The uniqueness of this study lies in the development of a new approach for mapping soil suitability based solely on large integral (LINTG) extracted from NDVI time series, which is a simple method for optimizing both time and financial resources [[29], [30], [31]]. For this purpose, this novel penological method is expected to be effective in detecting, mapping, and monitoring soil suitability without taking under consideration climate and anthropogenic actions. During last decades, several studies used vegetation indices derived from MODIS time series to detect soil quality [14,15]. In this context, numerous authors demonstrated that large integral (LINTG), one of phenological parameters, is a significant indicator of biomass production [[31], [32], [33]]. The novelty of this study is the development of a new approach for mapping soil suitability based solely on LINGT extracted from NDVI time series. It is a simple method for optimizing both time and financial resources to ensure good and efficient decision making, particularly in a world facing irreversible climatic and anthropogenic change. To demonstrate the effectiveness of this approach, a comparison with traditional methods is required. We used the MCDA method including (slope, depth, water holding capacity and salinity) for this purpose, which necessitates a large amount of work based on sampling, laboratory analysis, and heavy processing and consumes a deal of time and resources compared to our method.

## 2 Materials

### 2.1 Study area

**Fig. 1:**
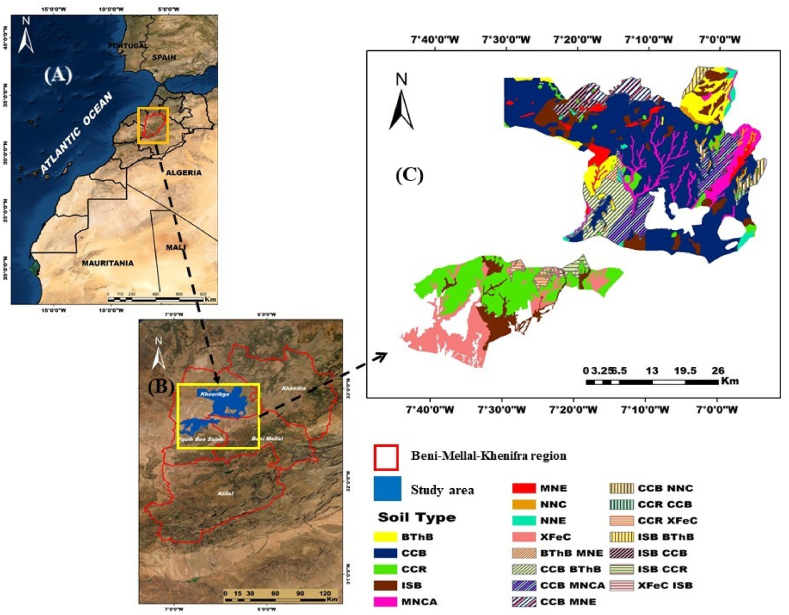
The study site's position where (A) refers the position acording the world, (B) sitution acording region,(C) stand for soil type.

The study area is situated in the central region of Morocco, in the Beni-Mellal-Khenifra region, which encompasses the provinces of Fkih Ben Saleh and Khouribga. The economy of this area is predominantly based on fallow agriculture due to the presence of fertile soils and water reserves. The study area covers a total land area ranging from 1541 km^2^ between latitudes 32° 30''0′N and 33° 40''0′N, and longitudes 6° 10''0′W and 7° 00''0′W, with an elevation varying between 440 and 882 m above sea level. The cultivated land accounts for 70 % of the total area, with cereals being the dominant crop, covering 42.7 % (Fig. 1,Table 1). Moreover, the Köppen classification, is a widely used system for classifying climates based on temperature and precipitation patterns. This classification system provides a framework for understanding and categorizing different climate types around the world. By analysing long-term climate data, including temperature and precipitation measurements, it's helps researchers, geographers, and scientists classify and describe the diverse climates found on our planet. It offers valuable insights into climate zones, aiding in the study of vegetation, agriculture, and other aspects influenced by climate conditions [34]. According to the Köppen classification the climate of the region is semi-arid to arid, with a dry season extending from April to October and a rainy season from November to March. The average annual rainfall is 350 mm, and the annual evaporation rate is approximately 1800 mm [34,35]. The annual temperature ranges from 3.5 °C in winter to 38 °C in summer, with an average temperature of 17 °C. The primary lithological formations found in the region include limestones and dolomites of the Lias, alluvium\colluvium, red clays of Trias, schists, sandstones, and quartzites from Palaeozoic [36].

**Table 1:** Description of the soil unit's codes.

Classes	Sub-classes	Groups	Codes
Raw mineral soils	no climatic	Erosion	MNE
Little evolved soils	no climatic	Erosion	NNE
Little evolved soils	no climatic	Colluvial contribution	NNC
Calci-magnesic soils	Carbonates	Rendzines	CCR
Calci-magnesic soils	Carbonates	Limestone browns	CCB
isohumic soils	In pedoclimate in the rainy season	Subtropical browns	ISB
Browned soils	Temperate humid climates	Browns	BThB
iron sesquioxide soils	Fersiallitics	Low-leaching calcium reserve	XfeC
Little evolved soils	no Climatic	Collu-alluvial contribution	NNCA

### 2.2 Soil data

The study team conducted field surveys from June to October 2000 and March to September 2022, with additional trips to identify the boundaries of soil units in areas with high levels of stones. Soil samples collected during the fieldwork were stored as they were obtained, with one profile out of every eight being taken, resulting in a total of 60 profiles. Soil analysis was carried out in the laboratory of the IAV Hassan II, using recognized methods adopted for Moroccan soils for pedological classification, including the CPCS (1967), except for isohumic soils, for which the Aubert (1965) classification was used. The distinction of soil units at the series level was based on depth. Stoniness and slope were taken into consideration during the agronomic classification. The categories of this classification are: class, subclass, group, subgroup, family, series (Table 1). The study area consisted of ten soil classes, with 5 % crude mineral soils, 14 % poor soils, 40 % calcium-magnesium soils, 19 % iso-humic soils, 6 % brown soils, 8 % iron sesquioxide soils, and the remaining soils classified as complex (Table .1).

### 2.3 Satellite data

To implement the RS-based phenological method, we utilized MODIS (Moderate Resolution Imaging Spectroradiometer) data to track the spatial and temporal changes in phenological parameters. Between 2007 and 2016, a collection of 240 images of the MOD13Q1 16-day composites product, with a resolution of 250 m, was obtained to cover the study area. This equates to an average of 24 images per year. All the images were acquired using the United States Geological Survey (USGS) reverb tool provided by NASA LP DAAC. The MOD13Q1 product is derived from the Level-2G daily surface reflectance gridded data, specifically the MOD09 and MYD09 8-day composites series. The Constrained View Angle-Maximum Value Composite method (CV-MVC) was employed in the calculation process. The MODIS-Terra satellite, operated by NASA, follows a near-polar orbit and is equipped with various spectral bands including NDVI, EVI, Blue, NIR, Red, and MIR, as well as quality bands [37]. The NDVI layers were particularly utilized to generate NDVI time series data. For the purpose of monitoring phenological parameters, the NDVI index was selected due to its sensitivity in detecting changes in vegetation canopy, especially in areas with low plant density. This distinguishes it from other indices such as the Enhanced Vegetation Index (EVI) [33]. Additionally, we incorporated two vital factors (i.e., elevation and slope) by using a digital elevation model (DEM) at 30 arc-seconds obtained from NASA Shuttle Radar Topography Mission (SRTM).

## 3 Methodology

**Fig. 2:**
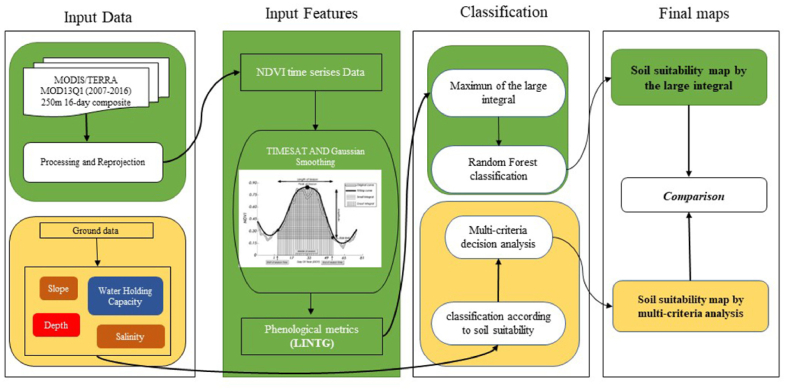
Workflow of the methodology for producing the Soil suitability.

To map soil suitability, two methods were evaluated and compared. The first one is based on several physico-chemical soil parameters (i.e., slope, soil salinity, depth, and soil water holding capacity), while the second one is based on the phenological behaviour of vegetation cover which represents the overall plant productivity. Fig. 2 summarizes the main methodological steps for soil mapping that include processing, classifications techniques, and mapping.

### 3.1 Geographic information system-multi-criteria decision analysis (GIS-MCDA)

By assessing the overall performance of various input decision alternatives, the MCDA technique can yield an optimal solution that takes into account the uncertainties linked to evaluating criteria [1]. MCDA methodologies can be categorized as either multi-objective or multi-attribute methods, and their main focus is on developing approaches to merge multiple criteria into a single outcome [38]. In this study, we adapted a GIS-based method of analysis by combining MCDA and GIS due to their strengths appear to complement each other. This strategy exploits the advantages of the GIS as a powerful and integrated tool with exclusive functions for storing, analysing/displaying georeferenced data, and the MCDA approach, as a rich set of algorithms and methods for designing, assessing, ordering criteria, and alternatives [6,39]. It is necessary to determine the criteria attributes in light of the specific circumstances being evaluated. The chosen set of criteria must effectively mirror the decision-making context and play a role in determining the ultimate outcome. To create a map of soil suitability, a process of evaluating multiple criteria is necessary. The characteristics indicating soil suitability must be obtained from both spatial and non-spatial data under various conditions [1,40]. The soil classification system utilized in this study is the Adjusted Gross Revenue (AGR) system, which consists of four classes. This system was cross-referenced with the Food and Agricultural Organization (FAO) classification [5,41] to assess soil suitability. The suitability ratings were determined based on soil characteristics, ranging from highly suitable to not suitable [42,43]. We relied only on consistent criteria that are naturally conditioned by paedogenetic processes, such as slope, depth, and water holding capacity (depend on the texture and depth). Fertility parameters were not considered in this study because they vary from one season to another and during the same agricultural season given the nature of the crops cultivated and the amendments made by the farmers [43]. Four criteria are considered as input for the soil suitability mapping which are: soil depth, soil water holding capacity, slope and salinity.

#### 3.1.1 Soil depth

**Fig. 3:**
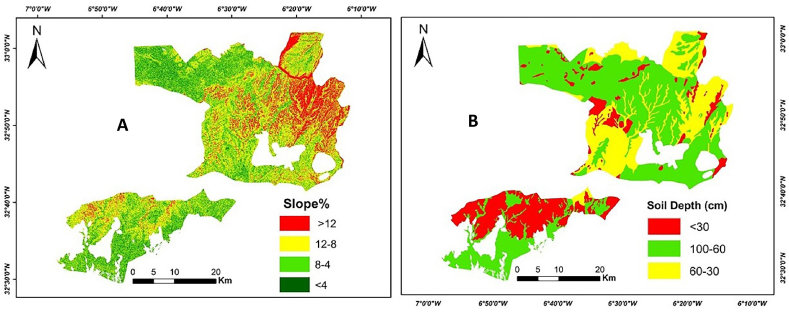
Maps of soil parameters; Slope (A) and Soil depth (B).

Soil depth is the most important parameter for mapping soil suitability [44,45]. Soil depth influences water availability. For every crop, it is essential to have a soil depth that is sufficient to offer the necessary physical support for the aboveground parts of the plant [1]. The growth of vegetation and root penetration, as well as its soil protective properties, are also impacted. In the study area, the depth of the soil varies between 10 and 100 cm as shown in Fig. 3. The area more than 100 cm are considered as very suitable area (S1), the areas with between 100 and 60 cm are considered as suitable areas (S2), the areas between 60 and 30 cm are considered as moderately suitable areas (S3) and the areas less than 30 cm are considered as unsuitable areas NS (Table 2).

**Table 2:** Soil suitability criteria [43].

	Soil parameters			
soil suitability classes	Depth (cm)	Slope%	Capacity of store water(mm)	Salinity(mmhos/cm)
S1 (Very suitable)	≥100	<4	≥150	<2
S2 (suitable)	100–60	4–8	110–150	2–4
S3 (moderately suitable)	60–30	8–12	75–110	4–8
NS (not suitable)	>30	>12	<75	>8

#### 3.1.2 Soil water-holding capacity

Soil water-holding capacity is the amount of water that a given soil can hold for crop use [45], which is essential for studying the response of vegetation and hydrologic system [46]. The ability of a soil to hold water for crop use is determined by several factors, including soil texture, soil depth, field capacity (FC), and permanent wilting point (PWP). Soils with smaller particle sizes, such as silt and clay, have a greater surface area, which affects their soil texture and water-holding capacity. To determine field capacity (FC) and permanent wilting point (PWP), we used the equations developed by Baize D et al.1995 [47]. It is based on the textural characteristics of the soil. Eq. (1) and Eq. (2); the soil water holding capacity classes map of the study shows that the area with more than 150 mm is considered as very suitable area (S1). The area with a slope between 110 and 150 mm is considered as a suitable area (S2), the area between 75 and 110 mm is considered as moderately suitable area (S3) and the area less than 75 mm is considered as not suitable area NS (Table 2).To determine the water holding capacity of the soil (3), we used equations (1), (2)) [43]:

(1) FC(mm)=43.638−0.31×(%sand)

where FC is taken as the existing water content held in the soil after 48 h of drainage.

And

(2) PWP(mm)=−0.83+0.77×(%clay)−0.0054

where PWP corresponds to the soil moisture from which the plant cannot take water.

From equations (1), (2) we have determined the water holding capacity (WHC) as:

(3) WHC=(FC–PWP)×D×AD

where D and AD are the depth and the apparent density.

#### 3.1.3 Slope

The slope is an angle that is created between a horizontal reference plane and any point on the earth's surface [48]. In the GIS analysis, the slope identifies the steepness at each cell of a raster surface [49] The degree of slope negatively affects irrigation and mechanization practices [14]. Nevertheless, as the slope becomes steeper, the risk of erosion also increases, which results in the depletion of organic matter and nutrients from the soil. For these reasons, it has been considered as a criterion for soil suitability assessment in this study. The slope layer was calculated in percent using a digital elevation model (DEM) with a spatial resolution of 30 m. The slope's classes map of the study area is shown in Fig. 3. The area less than 2–4% is considered as very suitable area (S1); the area with a slope between 4 and 8% is considered as suitable area (S2); the area with a slope of 8–12 % is considered as moderately suitable area (S3); the area more than 12 % is considered as no suitable area NS (Table 2).

After classifying the soil parameters according to the soil suitability classes, we found that salinity and water holding capacity are not constraints affecting the soils in the study area (Fig. 3).

### 3.2 Suitability modelling

The final step was to combine the various soil's factors to identify the most suitable areas for agriculture. To meet the following challenges, a model was constructed to match the environmental requirements. Suitability rankings from 1 to 4 and the associated threshold values for each criterion are shown in Table 2 and were assigned to the classes within each map layer to facilitate the suitability analysis. All the spatial data were converted to a vector form. In the next step, we assign automatically the suitability class for each polygon according to the aforementioned criterion using a program developed in Visual Basic Applications by Ref. [43]. Finally, a soil suitability map was generated and divided into four classes including, highly suitable (S1), moderately suitable (S2), marginally suitable (S3), and unsuitable (NS) (Fig. 6).

### 3.3 The accuracy of vegetation information derived from MODIS time series data

Several researchers evaluated the potential of MODIS imagery for evaluating soil properties [15,50]. They achieved this by establishing statistical correlation models between sixteen spectral indices and nine principal component analysis (PCA) components from MODIS bands. Despite the fact that the spectral indices for vegetation were not included, the findings indicated that statistical models of MODIS bands could accurately estimate soil properties using spectral indices of soil reflectance. Additionally, MODIS data can be acquired daily, 4-Day, 8-Day, 16-Day, monthly, quarterly, yearly, and also used to create time series data for a single year or spanning several years. The high temporal density of MODIS time series data can reduce noise introduced by other dynamic factors such as variations in management [22].

### 3.4 Suitability map based on phenological parameters

**Fig. 4:**
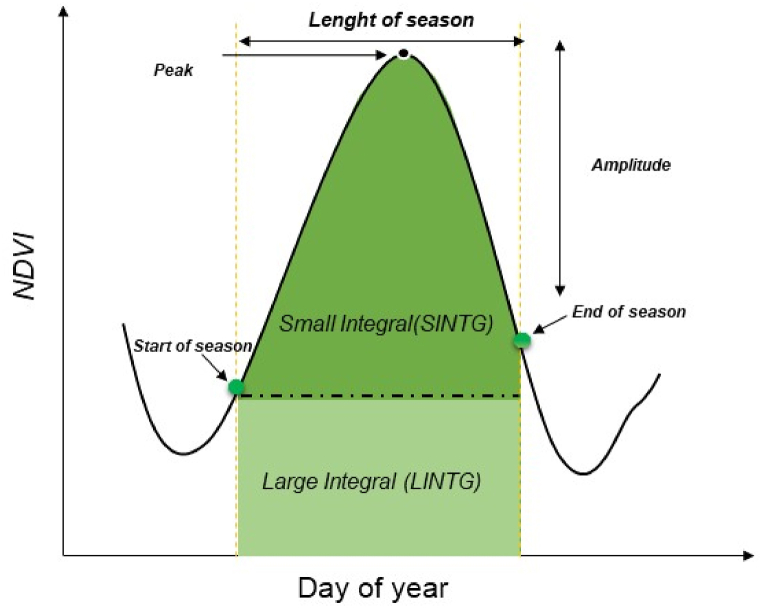
Some Phenological parameters of the vegetation season.

The NDVI time series profiles generated from 2007 to 2016 using the TIMESAT software were used to analyze and extract the phenological parameters [51]. The Gaussian Asymmetric filter was chosen due to its low sensitivity to noise and its large ability to smooth time series data [50,52]. Certain phenological parameters of the plant activity from the modelled curve can determined after the smoothing and construction of the smoothed NDVI time series (Fig. 4) [33,53]. In total 13 phenological parameters can be extracted with TIMESAT (Table 3). In this study, the large Integral (LINTG) has been chosen among other metrics to employ for the period between 2007 and 2016, as a good indicator biomass production of vegetation cover and to exclude biotic and abiotic factors except those linked to soil potential and suitability [31,33]. Furthermore, the maximum values of average annual LINTG of 10 years from 2007 to 2016 were obtained as a single layer.

**Table 3:** Seasonality parameters in TIMESAT.

Phenological Parameters	Abbreviation	Description
Start of season	SOS	Time for which the left edge has increased to 10 % of the seasonal amplitude measured from the left minimum level.
End of season	EOS	Time for which the right edge has decreased to 10 % of the seasonal amplitude measured from the right minimum level.
Middle of season	MOS	Mean value of the times for which the left part of the VGI curve has increased to the 90 % level and the right part has decreased to the 90 % level.
Length of season	LOS	Time from the start to the end of the season.
Base value	BVAL	The average of the left and right minimum values.
Maximum value	PEAK	Maximum VGI value for the fitted function during the season.
Amplitude	AMPL	Difference between the peak value and the base level.
Large integral	LINTG	The area under the smoothed curve between SOS and EOS.
Small integral	SINTG	The area below the base level from the SOS to EOS.
Left derivative	LDERIV	Rate of increase at the SOS between the left 10 % and 90 % of the amplitude.
Right derivative	RDERIV	Rate of decrease at the EOS between the right 10 % and 90 % of the amplitude.
Start of season value	SOSV	Start of season value.
End of season value	EOSV	End of season value.

### 3.5 Classification of the maximum of the large integral

In the present study, Random Forest (RF) algorithm was used as a classification method. The RF algorithm is a machine learning technique that combines multiple decision trees to perform both classification and regression tasks. Each tree in the forest is built using a random subset of the input features, ensuring that each tree has a unique perspective on the data. As the number of trees in the forest increases, overfitting is reduced and the generalization error is improved. The accuracy of the forest is dependent on the strength of individual trees and their correlation. To reduce inaccuracy, the algorithm selects a random subset of features for each node in the tree. The model's response to an increase in the number of variables is also estimated to ensure the relevance of the features used in the analysis [[54], [55], [56]]. Finally, soil suitability map was generated and divided into four classes including, highly suitable (S1), moderately suitable (S2), marginally suitable (S3), and unsuitable (NS).

## 4 Results

### 4.1 Soil suitability map based on physico-chemical parameters of soils

**Fig. 5:**
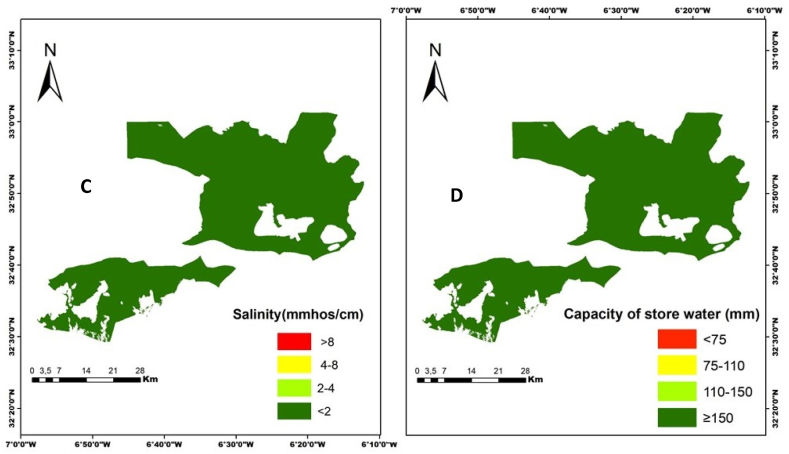
Final maps of soil parameters; Salinity (C) and soil Water holding capacity (D).

**Fig. 6:**
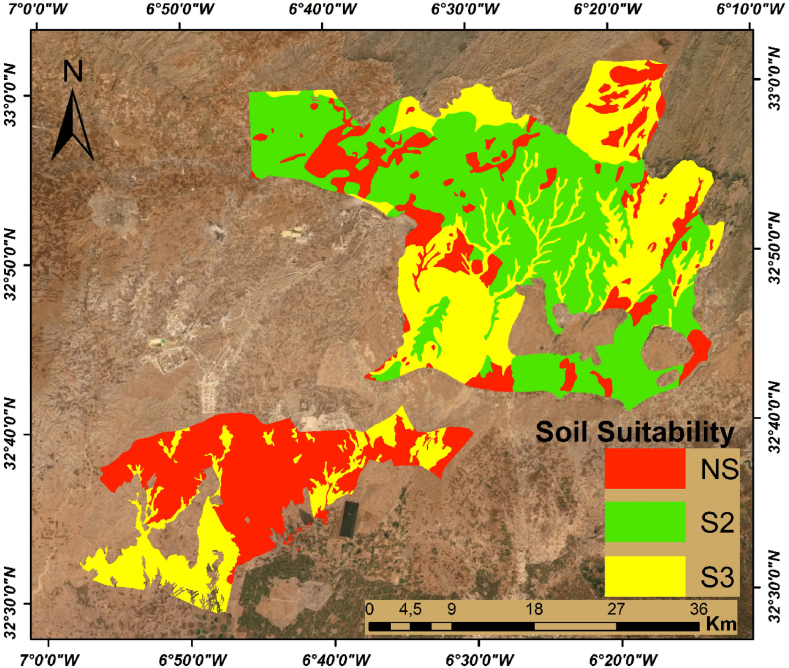
Soil suitability map of physical-chemical parameters of soil where (S2: suitable soil, S3: marginally suitable, NS: unsuitable soil).

The soil suitability map has been established by the combination of the pre-discussed factors that characterize soils (salinity, depth, slope, and water-holding capacity). We obtained 3 soil suitability classes (S2: suitable soil, S3: marginally suitable, NS: unsuitable soil) (Fig. 6). For the class S1 is not detected by the MCDA method. The suitable areas are located north of the study area. The unsuitable and moderately fit areas are located mainly in the south. Class S2 is the suitable class which is generally explained by the combination of a significant depth (60 cm and 100 cm), a low slope (2 % and 8 %), a high value of water holding capacity, and low salinity (Fig. 5). In addition, This and according to measurements and field knowledge, these soils are characterized by a lighter texture. Moreover, Soils of class NS (not fit), it is the combination of a very high slope (higher than 12 %) and a smaller depth (less than 30 cm). We can conclude that the most important factors in the mapping of soil suitability are soil depth and slope.

### 4.2 Suitability map based on large integral

**Fig. 7:**
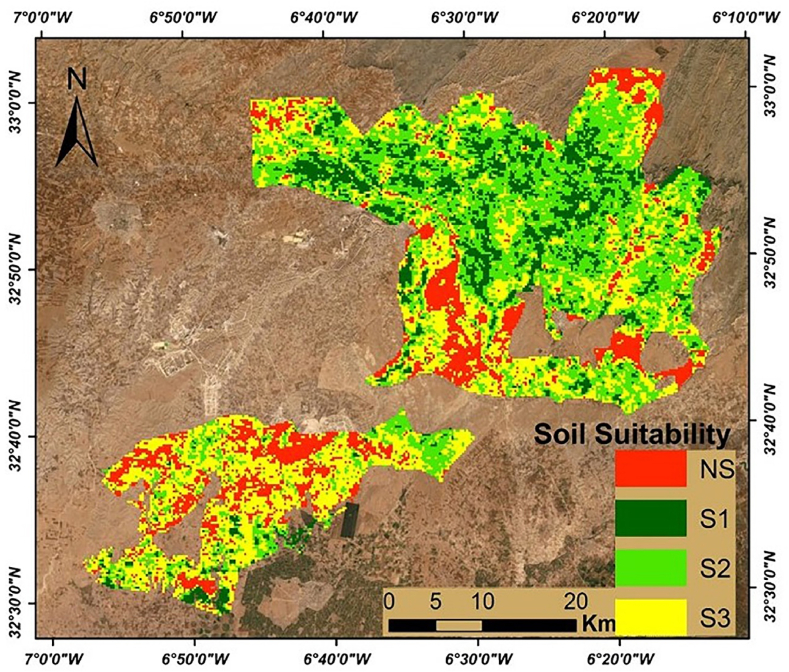
Soil suitability map based on LINTG where (S1: very suitable, S2: Suitable soil, S3: marginally suitable, NS: unsuitable soil).

The soil suitability mapping was performed based on the maximum of the large integral (LINTG) for a period of 10 years (2007–2016) derived from the time-series NDVI phenological parameter. Indeed, this parameter showed large spatial variability. This production is related to the soil's type and their vegetation production potential. Where NDVI values less than 0.3 indicate low biomass production, NDVI values that are between 0.30 and 7 indicate moderate biomass production, and values between 7.5 and 8.2 indicate high biomass production. Based on these results, the large integral is considered as one of the key parameters for discriminating different classes of soil suitability (Table 4). The specific objective of this step is to classify the large integral to identify the different classes of soil suitability (Fig. 7). The classification has been established to compare the results of the classification with the results of physico-chemical parameter data in terms precision. LING was classified into 4 classes (S1, S2, S3 and NS). The highest potential of production class S1 was observed on the irrigated area and the crop a high productivity; this class is not classified in the method based on the soil parameters (Figs. 6 and 7).

**Table 4:** Classes of biomass production in the large integral (LINTG) [33].

NDVI values	Biomass production potential
<0.3	Low biomass production
0.3–7.5	Moderate biomass production
7.5–8.2	High biomass production
>8.2	Very high biomass production

### 4.3 Statistic analysis comparing phenological approach and MCDA approach

To compare the number of the pixel in two methods, identify the correlation between different soil suitability classes and represent how correlations change from one class grouping to another, we apply the cross-tabulation method to quantitatively analyze the relationship between multiple classes. Statistical analysis of raw data was used to find patterns, trends, and probabilities [57]. Numerous studies propose that cross-tabulation is a highly favoured technique for analyzing survey and market research data [58]. According to qualtrics, cross-tabulation analysis and frequency analysis of single variables account for over 90 % of all research analyses [59,60]. In this study, we use the soil suitability map large Integral (LINTG) to analyze changes in soil suitability map from MCDA. In this case, we have two categorical rasters with integer values corresponding to different soil suitability classes. The first two columns of the table show, respectively, MCDA suitability class and phenological suitability classes. The third column shows the number of pixels (frequency) (Table 6). In cases where values for both column 1 and 2 coincide, no MCDA suitability class transition occurred. On the opposite side, different values are evidence of changes.

**Table 5:** Determination of percentage of soil suitability class using for the two methods.

Soil suitability classes	MCDA approach	Phenological approach
NS	47,15	19,57
S3	24,39	39,89
S2	28,45	34,94
S1	0	5,57

**Table 6:** The contingency table.

MCDA SUITABILITY	PHENOLOGICAL SUITABILITY	Freq
S2	NS	182
S2	S3	578
S2	S2	1142
S2	S1	543
NS	NS	416
NS	S3	779
NS	S2	471
NS	S1	181
S3	NS	411
S3	S3	788
S3	S2	774
S3	S1	228

**Fig. 8:**
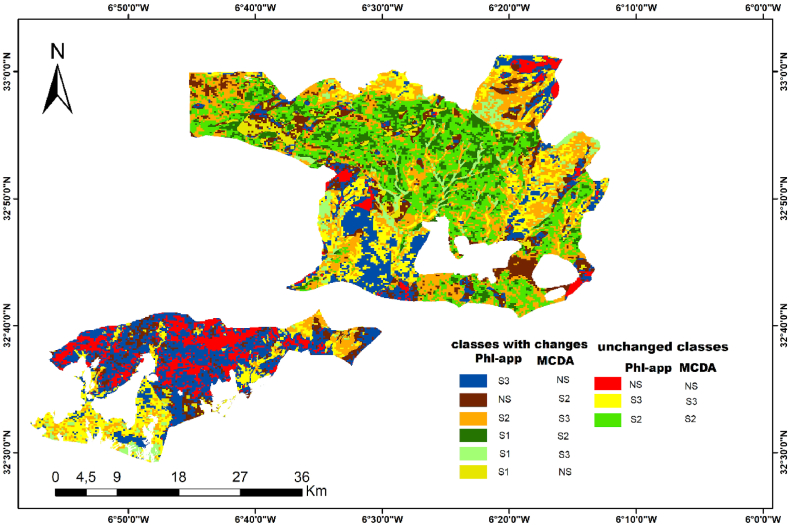
Map resulted from cross-tabulation classification of two suitability maps where (S1: very suitable,S2: suitable soil, S3: marginally suitable, NS: unsuitable soil).

We can also convert the contingency table into a confusion matrix where the confusion matrix is easier to analyze than the contingency table (Table 6). From the contingency table and matrix (Fig. 7), we can evaluate how many pixels remained in the same class, and how many classes converts to another class. If we look closely, we see that several areas of NS have moved to the classes S3 and S2, and several of the areas S2 and S3 have moved to the class S1 represented the fallow and irrigated area with high productivity, which proves the appearance of S1 class (Fig. 8).

## 5 Discussion

**Fig. 9:**
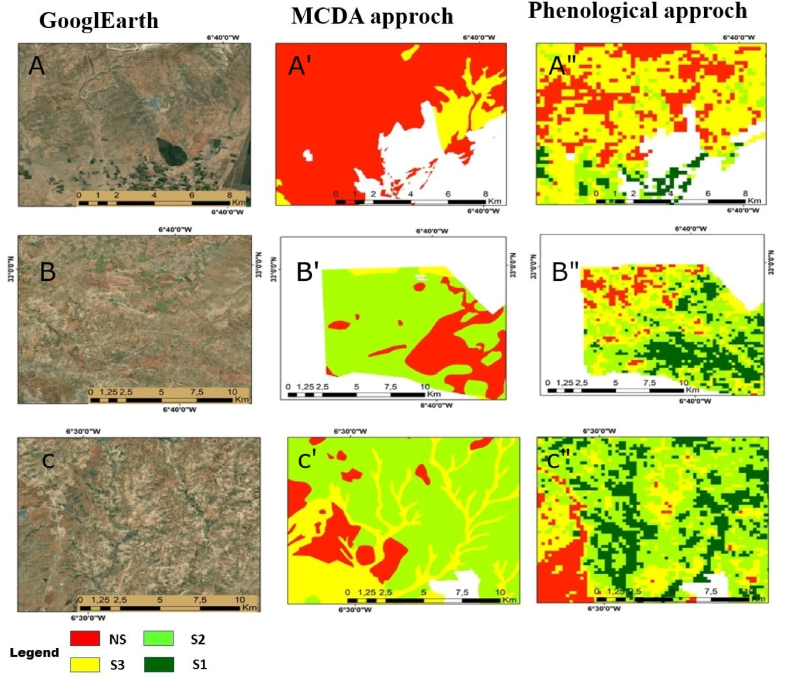
A visual comparison of results for a zoomed area using Google Earth (A, B, C), MCDA (A′, B′, C′), and Phenological approach (A″, B″, C″). Where S1 (very suitable), S2 (suitable soil), S3 (marginally suitable), NS (unsuitable soil).

To ensure sustainable agricultural production, it is necessary to analyze the suitability of soil. The Food and Agricultural Organization (FAO) has recommended an approach to evaluate soil suitability for vegetation production based on climatic, terrain, and soil properties. However, assigning an area to a suitability class can be challenging due to variations in soil properties within the area and the matching of soil properties with multiple suitability classes [[61], [62], [63]]. The advancement in GIS and multi-criteria methods has modified and improved this approach. GIS allows for the capture, storage, manipulation, analysis, and presentation of spatial or geographic data. It provides a powerful framework for organizing and visualizing complex information, enabling researchers to gain valuable insights and make informed decisions [5,38]. The integration of multi-criteria methods within GIS further enhances its capabilities by incorporating multiple factors and criteria into the decision-making process [21]. By incorporating multi-criteria methods into GIS, researchers can effectively analyze spatial data while considering multiple factors simultaneously [19,64].The integration of GIS and multi-criteria methods has led to significant improvements in a wide range of applications. Numerous studies have explored and validated the effectiveness of this integrated approach. For instance Ref. [65], conducted a study on land-use suitability analysis using GIS and multi-criteria evaluation methods. However, MCDA has some limitation such as data availability and quality: MCDA relies on the availability and quality of data for criteria and alternatives. In situations where data is scarce or unreliable, the accuracy and reliability of the analysis may be compromised. Furthermore, Inability to capture dynamic changes: MCDA often assumes a static environment, ignoring temporal changes and dynamics. It may not adequately address evolving conditions, trends, and uncertainties that can impact decision-making over time. Moreover, Difficulty in comparing incommensurable Criteria: MCDA assumes that criteria are commensurable, meaning they can be measured and compared on the same scale [17,66]. In this context, the use of remote sensing techniques can overcome the limitations of classical soil suitability mapping by providing an alternative method across various spatio-temporal scales. This can be achieved by using satellite-derived vegetation response to soil, climate, and agricultural techniques’ conditions, which offers a potential alternative proxy of soil suitability [[23], [24], [25],^62^]. In this study, the phenological approach has successfully mapped the S1class (very suitable), which represents 5.57 % of the total area (Table 5). However, this class was not identified in the multi-criteria method. Field information suggests that these areas have very suitable soil, and the only limitation is water resources that can be overcome through irrigation. The S3 class, covering 24.39 % of the total area, was identified in the phonological map, with a decrease of almost 20 % in the non-suitable area compared to the multi-criteria method. The phenological-based approach classified less than 19.57 % of the total study area as unsuitable, whereas the multi-criteria method generated a 47.15 % unsuitable area (Table 5). The outcome can be clarified by the fact that this classification is sensitive to changes in the climate, such as an increase in extreme weather events, such as precipitation and temperature, which primarily affect arid and semi-arid [31,35]. In contrast, the penological method aims to address all climate change requirements by utilizing the maximum of the large integral for 10 years to eliminate all obstacles and preserve only soil suitability. Fig. 9 illustrates the results of both techniques, and their suitability classes were evaluated by comparing them to ground truth. Through visual comparison, it was discovered that the large integral method is more accurate because it indicates that the soil is not suitable for agriculture in areas with phosphate mining activities in the study area, while the direct method indicates that the soil is suitable for cultivation in these locations. In the second part of the study, there was a notable difference in the results between the direct and indirect methods. The direct method indicated that the soil was not suitable for vegetation production, whereas the large integral showed a high potential for vegetation production and identified existing irrigated areas, indicating the high suitability of these areas for intensive vegetation production [35].

The third part of the study revealed that the indirect method was more accurate and closer to the ground reality than the direct method. The use of spatial remote sensing proved to be an effective tool for identifying and monitoring soil suitability. It enables the differentiation of various classes of soil suitability based on the size of the large integer, which is directly related to the crops' integrated state, absorbed photo-synthetically active radiation, and annual net primary productivity. It also facilitates the mapping of inaccessible areas, reducing the need for costly and time-consuming surveys. Several studies have demonstrated the significant correlation between soil suitability and climatic parameters such as temperature and precipitation. The soil resource is an essential aspect of sustainable agriculture, recognized as a crucial agronomic variable that entirely relies on the food production of specific agricultural land, ensuring an increase in food demand [49,67,68]. The study has shown the reliability of using low spatial resolution data for large-scale analysis. Although the results were satisfactory, the accuracy could be further improved by using new satellite sensors and the synergy of remotely sensed data from multiple sensors that provide hyperspatial, hyperspectral, and hyper-temporal observations. Establishing a classification of soil suitability in the study area is possible using the obtained parameters, forming the basis for several in-depth studies. This comparison between the direct and indirect methods demonstrates the potential of remote sensing as a decision-making tool for mapping soil suitability.

## 6 Conclusion

The process of mapping soil suitability is crucial in determining land use based on its potential and safeguarding natural resources for future generations. Thus, there is a pressing need to create quick and dependable methods for detecting and mapping areas suitable for various purposes. To facilitate decision-making, it is essential for authorities and planners to have reliable estimates of suitable areas for agricultural locations. The predictive model described in this context, developed using the latest data-processing techniques, can support such endeavours. In this study, identification of suitable areas for agricultural land by comparing too methods at regional scale carried out in the Khouribga Region. In light of study results, method considers the factors that characterize soils using MCAD to produce the soil suitability map. It makes it possible to map and spatialize the suitability of soils, namely S2 (suitable soil), S3 (moderately suitable soil), and NS (non-suitable soil). The second method, which depended on the phenological parameters of the NDVI time series, was able to determine 4 soil suitability classes: S1 (very suitable soil), S2, S3, and NS. Therefore, the phenological analysis provides information to deepen our understanding of the spatio-temporal variability of land surface phenology in arid and semi-arid area. In perspective, assessment of environmental, agronomic and socio-economic consequences of phenological changes can improve the awareness of stakeholders to adapt it to take decisions to limit the impacts of change on ecosystems and society. The results demonstrated the ability of phenological parameters to identify and monitor the main soil suitability classes in the study area.

## Funding

This research was funded by Researchers Supporting Project number (RSP2023R351), 10.13039/501100002383King Saud University, Riyadh, Saudi Arabia.

## Data availability statement

The data that support the findings of this study are openly available in https://lpdaac.usgs.gov/products/mod13q1v006/at https://doi.org/10.5067/MODIS/MOD13Q1.006. The other soil data that support the findings of this study are available on request from the corresponding author. The data are not publicly available.

## CRediT authorship contribution statement

**Maryem Ismaili:** Writing – review & editing, Writing – original draft, Validation, Software, Resources, Data curation, Conceptualization. **Samira Krimissa:** Writing – review & editing, Validation, Supervision. **Mustapha Namous:** Writing – review & editing, Visualization, Validation, Supervision. **Kamal Abdelrahman:** Writing – review & editing, Investigation, Funding acquisition. **Abdelghani Boudhar:** Writing – review & editing, Visualization. **Mohamed Edahbi:** Writing – review & editing, Methodology, Formal analysis, Data curation. **Youssef Lebrini:** Writing – review & editing, Software, Resources, Formal analysis, Conceptualization. **Abdelaziz Htitiou:** Validation, Software, Methodology, Formal analysis, Data curation. **Soufiane Maimouni:** Supervision, Data curation. **Tarik Benabdelouhab:** Writing – review & editing, Visualization, Validation, Supervision, Project administration, Methodology.

## Declaration of competing interest

The authors have no relevant financial or non-financial interests to disclose and declare no Interest Statement.
